# The 38K-Mediated Specific Dephosphorylation of the Viral Core Protein P6.9 Plays an Important Role in the Nucleocapsid Assembly of Autographa californica Multiple Nucleopolyhedrovirus

**DOI:** 10.1128/JVI.01989-17

**Published:** 2018-04-13

**Authors:** Qingying Lai, Wenbi Wu, Ao Li, Wei Wang, Meijin Yuan, Kai Yang

**Affiliations:** a State Key Lab of Biocontrol, Sun Yat-sen University, Guangzhou, China; University of California, Irvine

**Keywords:** baculovirus, 38K, P6.9, HAD, phosphatase, AcMNPV, nucleocapsid assembly

## Abstract

Encapsidation of the viral genomes, leading to the assembly of the nucleocapsids to form infectious progeny virions, is a key step in many virus life cycles. Baculovirus nucleocapsid assembly is a complex process that involves many proteins. Our previous studies showed that the deletion of the core gene *38K* (*ac98*) interrupted the nucleocapsid assembly by producing capsid sheaths devoid of viral genomes by an unknown mechanism. All homologs of 38K contain conserved motifs of the haloacid dehalogenase superfamily, which are involved in phosphoryl transfer. The requirements of these motifs for nucleocapsid assembly, confirmed in the present study, suggest that 38K may be a functioning haloacid dehalogenase. P6.9 is also encoded by a core gene (*ac100*) and is required for viral genome encapsidation. It has been reported that multiple phosphorylated species of P6.9 are present in virus-infected cells, while only an unphosphorylated species is detected in the budded virus. Therefore, whether 38K mediates the dephosphorylation of P6.9 was investigated. An additional phosphorylated species of P6.9 in *38K*-deleted or -mutated virus-transfected cells was detected, and the dephosphorylated sites mediated by 38K were determined by mass spectrometry. To assess the effects of dephosphorylation of P6.9 mediated by 38K on virus replication, these sites were mutated to glutamic acids (phosphorylation-mimic mutant) or to alanines (phosphorylation-deficient mutant). Studies showed that the nucleocapsid assembly was interrupted in phosphorylation-mimic mutant virus-transfected cells. Taken together, our findings demonstrate that 38K mediates the dephosphorylation of specific sites at the C terminus of P6.9, which is essential for viral genome encapsidation.

**IMPORTANCE** Genome packaging is a fundamental process in the virus life cycle, and viruses have different strategies to perform this step. For several double-stranded DNA (dsDNA) viruses, the procapsid is formed before genome encapsidation, which may require basic proteins that help to neutralize the nucleic acid charge repulsion to facilitate the compaction of the genome within the confined capsid space. Baculovirus encodes a small basic protein, P6.9, which is required for a variety of processes in the virus infection cycle. The phosphorylation of P6.9 is thought to result in nucleocapsid uncoating, while the dephosphorylation of P6.9 is involved in viral DNA encapsidation during nucleocapsid assembly. Here, we demonstrate that a haloacid dehalogenase homolog encoded by baculovirus core gene *38K* is involved in nucleocapsid assembly by mediating the dephosphorylation of 5 specific sites at the C terminus of P6.9. This finding contributes to the understanding of the mechanisms of virus nucleocapsid assembly.

## INTRODUCTION

The family Baculoviridae includes a diverse group of insect viruses with large, double-stranded, circular DNA genomes that are packaged in rod-shaped, enveloped nucleocapsids (1, 2). There are four genera of Baculoviridae: Alphabaculovirus (lepidopteran nucleopolyhedrovirus [NPV]), Betabaculovirus (lepidopteran granulovirus [GV]), Gammabaculovirus (hymenopteran NPV), and Deltabaculovirus (dipteran NPV) (3). The Alphabaculovirus genus fall into two phylogenetic clades, representing group I and group II NPVs (4). Autographa californica multiple nucleopolyhedrovirus (AcMNPV) is in the genus Alphabaculovirus (type species, Autographa californica multiple nucleopolyhedrovirus) and was the first baculovirus to have its genome completely sequenced (5). During infection of insect cells, most well-characterized baculoviruses undergo a biphasic virus life cycle with the production of two phenotypic virions: budded virions (BVs) and occlusion-derived virions (ODVs) (6). These two forms of virions have a common nucleocapsid structure and carry the same genetic information but differ in the origin and composition of their lipid envelopes (7, 8). The BV acquires its envelope from the plasma membrane which is modified with viral proteins by budding from infected cells and is required for spreading infection among cells and tissues (9), while the ODV obtains its envelope from virus-induced intranuclear microvesicles within a peripheral area, called the ring zone (RZ), in the nuclei of infected cells and plays a role in infecting the midgut epithelial cells of a host to initiate primary infection (10).

Baculovirus infection causes the nucleus to swell and form the virogenic stroma (VS), which is a nuclear compartment composed of homogenous fibrillar electron-dense mattes with electron-lucent intrastromal spaces (11). The VS is thought to serve as a molecular scaffold for the transcription and replication of viral DNA and the subsequent packaging of DNA and assembly of nucleocapsids (6). Studies have shown that the nucleocapsid morphogenesis of baculovirus begins with the formation of empty, nearly full-length capsid sheaths, and the nucleoprotein core is concentrated from the base to the apex of the capsid cylinder (12).

Genomes of DNA viruses are compacted and packaged into virus particles with small basic proteins encoded by the host or virus. Some viruses directly utilize host histones for DNA packaging, such as polyomaviruses (13, 14) and papillomaviruses (15). Some viruses encode their own DNA-packaging proteins, e.g., adenoviruses (16, 17) and white spot syndrome viruses (WSSVs) (18). Baculovirus encodes a protamine-like protein, P6.9, which is rich in arginine, serine, and threonine residues and is thought to bind and condense viral DNA for packaging into the capsid sheath (19). Protamine-like proteins belong to a large group of proteins that are involved in DNA binding, known as sperm nuclear basic proteins (20). Posttranslational modifications (PTMs) of these basic proteins may be involved in extensive cellular, epigenetic, and chromatin changes (21). Evidence suggests that newly synthesized P6.9 is transiently phosphorylated in infected cells prior to participation in nucleocapsid assembly (22). In our previous study, multiple phosphorylated species of P6.9 were identified in cells infected with wild-type (WT)-resembling virus vP6.9:HA, while only an unphosphorylated species was detected in BVs (23). The various phosphorylation states of P6.9 were reexamined by acetic acid-urea polyacrylamide slab gel electrophoresis (AU-PAGE) analysis, which showed that a ladder composed of 7 phosphorylated species (no. 1 to 7 in AU-PAGE) and 1 unphosphorylated species (no. 0 in AU-PAGE) of P6.9 was detected using samples derived from cells infected with the WT-resembling virus vPK1:FLAG; in addition, 22 phosphorylation sites in P6.9 were identified by mass spectrometry (MS) (24). These included serines, threonines, and arginines. The protein kinase PK1 encoded by baculovirus is responsible for hyperphosphorylation of P6.9, as the deletion of *pk1* resulted in abolition of the 3 slowest-migrating rungs of P6.9 species (no. 5 to 7 in AU-PAGE), which in turn regulated the maximal expression of viral very late genes and viral infectivity (24). In addition to PK1 specifically regulating the hyperphosphorylation of P6.9, other protein kinases of the host are thought to regulate the hypophosphorylation of P6.9 (24). Thus, the phosphorylation status of P6.9 may be critical in the life cycle of baculoviruses.

Encapsidation of the viral genomes, leading to the assembly of infectious progeny virions, is an essential step in the virus life cycle. During nucleocapsid assembly, P6.9 is dephosphorylated, and viral DNA is condensed and packaged with P6.9 to form a DNA-protein core in the capsid sheath (19, 25, 26). The phosphatases of the virus and/or host that are responsible for dephosphorylation of P6.9 have not yet been identified. Baculovirus 38K is encoded by a core gene found in all baculovirus (27), nudivirus (28, 29), and polydnavirus (30) genomes. It belongs to the 38K (baculovirus)/ROP9 (apicomplexa) family of the haloacid dehalogenase (HAD) superfamily (31) and has been shown to be essential for encapsidation of viral genomes during virus infection (32). Practically all members of the HAD superfamily possess four highly conserved sequence motifs, DxD, S/T, K, and DD/GDxxxD/GDxxxxD (33), which are also present in 38K homologs. The majority of the enzymes in this superfamily, e.g., phosphate monoester hydrolases (phosphatases) or phosphoanhydride hydrolase P-type ATPases (31), are involved in phosphoryl transfer. Thus, whether 38K dephosphorylates P6.9 during nucleocapsid assembly was investigated.

In the present study, by constructing 38K mutant viruses, we investigated the requirement of the HAD conserved motifs in 38K for viral propagation and nucleocapsid assembly. The phosphorylated species of P6.9 present in *38K*-deleted or HAD motif-mutated virus bacmid DNA-transfected cells were then examined, and one slower-migrating rung representing higher phosphorylation of P6.9 species (no. 8 in AU-PAGE) was detected compared to the results for the control virus. By using MS, we identified 5 phosphorylated sites at the C terminus of P6.9 that were dephosphorylated by 38K. To confirm that the interruption of nucleocapsid assembly in *38K*-deleted-virus-transfected cells was due to the inability of 38K to dephosphorylate P6.9, we generated a recombinant virus with phosphorylation-mimic sites in P6.9. Our study determined that 38K is involved in nucleocapsid assembly by mediating the dephosphorylation of P6.9.

## RESULTS

### Conservation analysis of 38K homologs.

**FIG 1:**
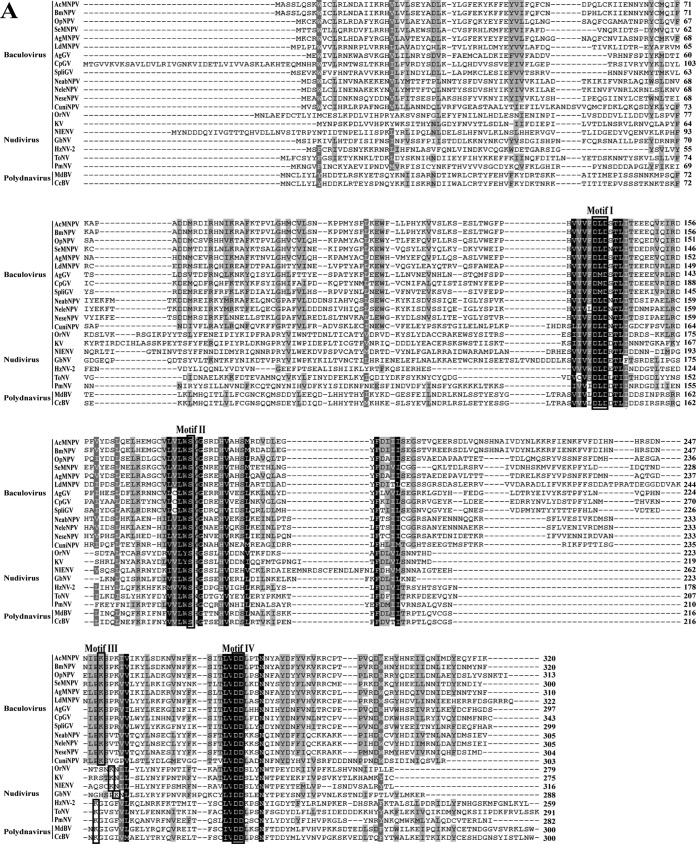

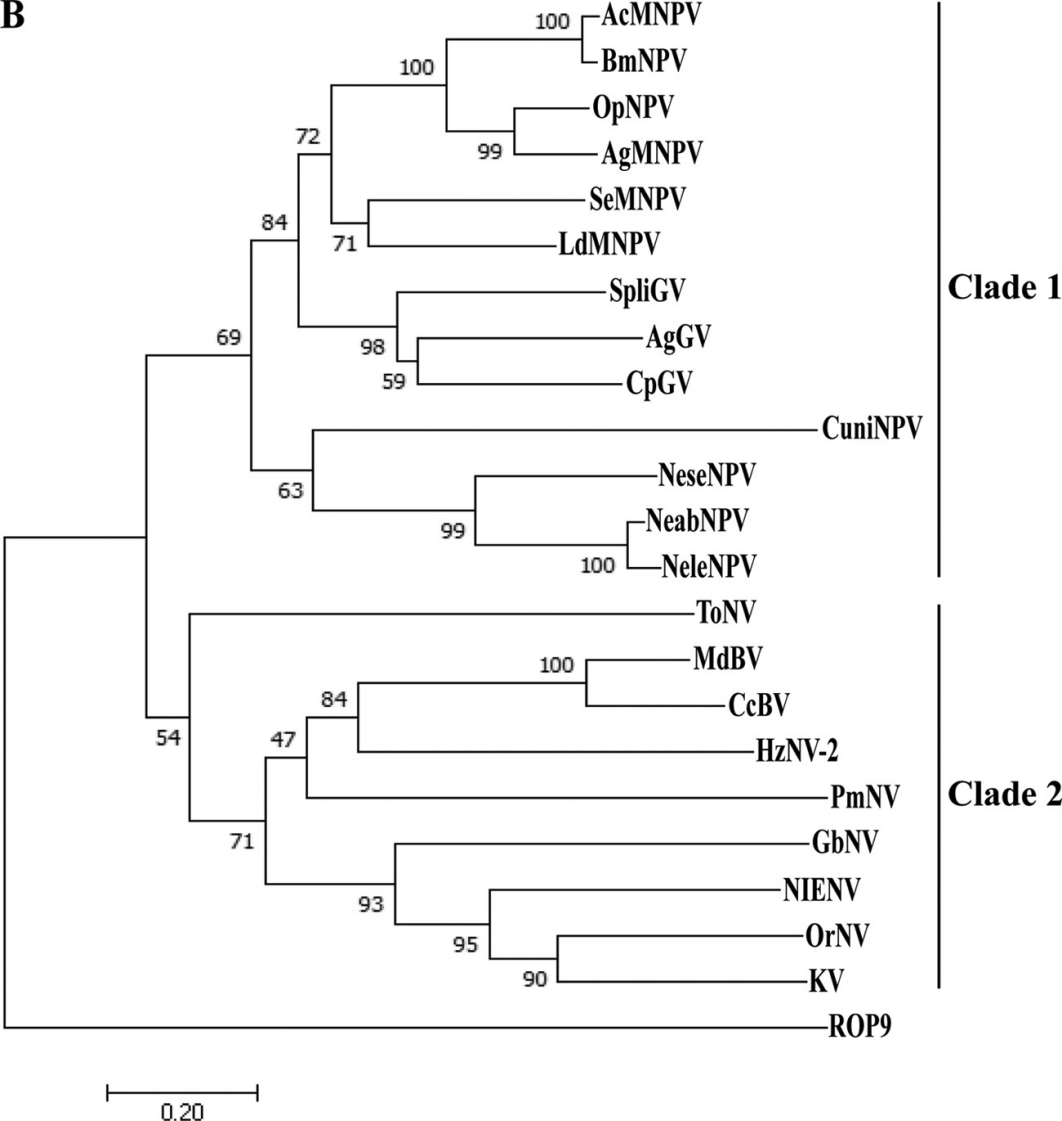
Bioinformatics analysis of AcMNPV 38K. (A) Sequence alignment of 38K homologs. The conserved amino acids of HAD motifs in 38K homologs are outlined by rectangles and indicated above of the sequence alignment. The following abbreviations are used, with GenBank accession numbers in parentheses: AcMNPV, Autographa californica multiple nucleopolyhedrovirus (NP_054128.1); BmNPV, Bombyx mori nucleopolyhedrovirus (AFN09011.1); OpMNPV, Orgyia pseudotsugata multiple nucleopolyhedrovirus (NP_046255.1); SeMNPV, Spodoptera exigua multiple nucleopolyhedrovirus (CDG72408.1); AgseNPV, Agrotis segetum nucleopolyhedrovirus (YP_529744.1); LdMNPV, Lymantria dispar multiple nucleopolyhedrovirus (ANS70986.1); AgseGV, Agrotis segetum granulovirus (YP_006267.1); CpGV, Cydia pomonella granulovirus (NP_148872.1); SpliGV, Spodoptera litura granulovirus (NP_258356.1); NeabNPV, Neodiprion abietis nucleopolyhedrovirus (YP_667907.1); NeleNPV, Neodiprion lecontei nucleopolyhedrovirus (YP_025256.1); NeseNPV, Neodiprion sertifer nucleopolyhedrovirus (YP_025256.1); CuniNPV, Culex nigripalpus nucleopolyhedrovirus (NP_203392.1); OrNV, Oryctes rhinoceros nudivirus (YP_002321398.1); KV, Kallithea virus (YP_009346005.1); NIENV, Nilaparvata lugens endogenous nudivirus (AHW98243.1); GbNV, Gryllus bimaculatus nudivirus (YP_001111268.1); HzNV-2, Helicoverpa zea nudivirus (YP_004956856.1); ToNV, Tipula oleracea nudivirus (YP_009116710.1); PmNV, Penaeus monodon nudivirus (YP_009051897.1); MdBV, Microplitis demolitor bracovirus (XP_008543854.1); CcBV, Cotesia congregate bracovirus (CAR82239.1). (B) Neighbor-joining phylogenetic analysis of 38K homologs performed by MEGA 7. ROP9, which belongs to the 38K/ROP9 family of the HAD superfamily, is encoded by Toxoplasma gondii (XP_002366872.1). The bootstrap scores of the nodes are shown.

Baculovirus 38K is encoded by a core gene found in all baculovirus (27), nudivirus (28, 29), and polydnavirus (30) genomes. Amino acid alignments showed that the C-terminal region of 38K homologs is more conserved than the N-terminal region (Fig. 1A). To obtain an overview of 38K evolutionary relationships, 38K homologs from baculoviruses, nudiviruses, and polydnaviruses were selected for phylogenic studies. The phylogenetic tree showed evidence for two clades: all the baculovirus 38K homologs belonged to clade 1, while clade 2 contained 38K homologs from nudiviruses and polydnaviruses (Fig. 1B), indicating that 38K homologs from nudiviruses and polydnaviruses are more distant from those from baculoviruses. A conserved domain search from the NCBI Conserved Domain Search databases (34) revealed that AcMNPV 38K belongs to the viral phosphatase superfamily. The region from amino acid (aa) 135 to 288 of AcMNPV 38K hits the IIIC subfamily of the HAD superfamily, which contains the enzymes involved in phosphoryl transfer (31). The characterized proteins within subfamily III are all phosphatases with four characteristic sequence motifs, DxD, S/T, K, and DD/GDxxxD/GDxxxxD, that are present in members of the HAD superfamily (35, 36). The results of the multiple-sequence alignment showed that all the 38K homologs in the families Baculoviridae, Nudiviridae, and Polydnaviridae contain the HAD motifs; specifically, they are D^140^xD^142^, S^177^, K^251^, and D^275^D^276^ in AcMNPV 38K (Fig. 1A). These results suggested that 38K may function as a viral phosphatase.

### The HAD motifs of 38K are essential for nucleocapsid assembly.

**FIG 2:**
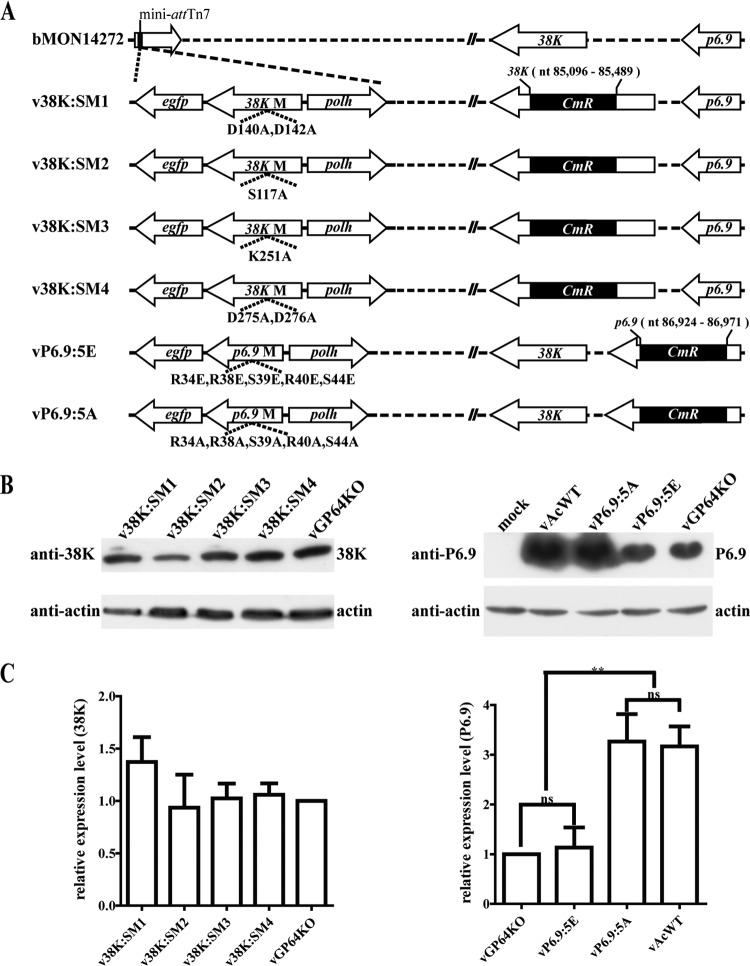
(A) Schematic diagram of the recombinant viruses used in this study. 38K-mutated viruses were constructed by mutating the amino acids of HAD motifs with alanines, and mutants of *38K* as well as the *polh* and *egfp* genes were transposed into the mini-*att*Tn7 locus of b38KKO individually. vP6.9:5E or vP6.9:5A was generated by changing the indicated amino acids of P6.9 to glutamic acids or alanines, respectively, and then the *p6.9* mutant together with *polh* and *egfp* were transposed into bP6.9KO. (B and C) Expression of 38K or P6.9 in recombinant virus-transfected cells. Sf9 cells were transfected with bacmid DNA of v38K:SM1, v38K:SM2, v38K:SM3, v38K:SM4, or vGP64KO to examine the expression levels of modified 38K or wild-type 38K (left panels) or transfected with vAcWT, vP6.9:5A, vP6.9:5E, or vGP64KO to examine the expression levels of modified P6.9 or wild-type P6.9 (right panels). At 48 h p.t., the transfected cells were collected and subjected to SDS-PAGE for immunoblotting with the anti-38K polyclonal antibodies (B, left panel) or with the anti-P6.9 polyclonal antibodies (B, right panel). Actin was detected with an antiactin monoclonal antibody as a loading control. The protein expression levels were analyzed by Gel-Pro analyzer 4 and normalized to the expression levels of actin (C). The differences between the protein expression levels were analyzed by Student's *t* test with GraphPad Prism software. Bar heights represent the average from three independent experiments, and the error bars indicate the standard deviations. **, *P* < 0.01.

To investigate the function of the HAD motifs in 38K during the virus life cycle, four recombinant viruses, v38K:SM1, v38K:SM2, v38K:SM3, and v38K:SM4, were generated, in which the HAD motifs in 38K were individually mutated to alanine residues (Fig. 2A).

**FIG 3:**
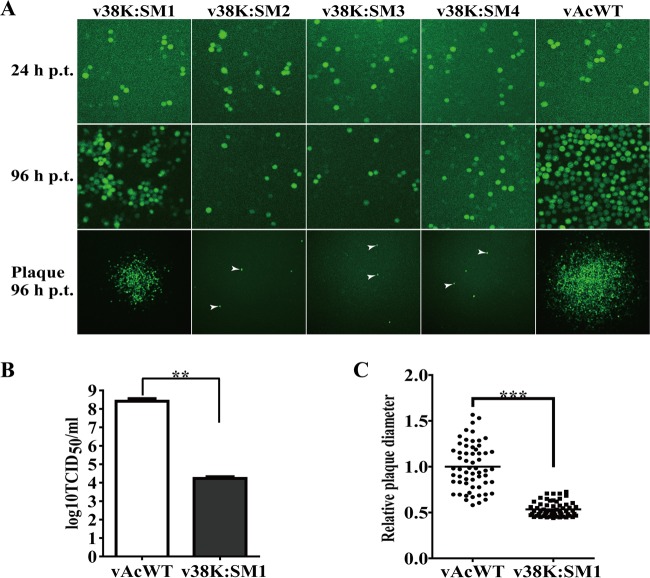
Analysis of virus replication and BV production in Sf9 cells. (A) Sf9 cells were transfected with 1 μg bacmids of the indicated recombinant viruses and monitored for infection by fluorescence microscopy as indicated by GFP expression. The top panels show cells transfected with bacmids at 24 h p.t., and the middle panels show the progression of virus infection at 96 h p.t. The bottom panels show viral plaques visualized at 96 h p.t. Arrowheads indicate the plaques formed by single cells. (B) Infectious BV production by vAcWT and v38K:SM1. Sf9 cells were transfected with bacmids of vAcWT or v38K:SM1. The supernatants of transfected cells were harvested at 96 h p.t., and the BV titers were determined by TCID_50_ endpoint dilution assay. Student's *t* test analysis of variance was carried out with GraphPad Prism software. Bar heights represent the average from three independent experiments, and the error bars indicate the standard deviations. (C) Plaque diameters in vAcWT- or v38K:SM1-transfected cells. A total of 60 plaques were measured in vAcWT- or v38K:SM1-transfected cells. Plaque size differences were analyzed by Student's *t* test with GraphPad Prism software. Bars indicate the medians for samples. **, *P* < 0.01; ***, *P* < 0.001.

Sf9 cells transfected with bacmid DNA of the 38K mutant viruses or the vAcWT (WT AcMNPV vAc^PH-GFP^) were monitored by fluorescence microscopy, since all viruses could express the *enhanced green fluorescence protein* (*egfp*) gene. At 24 h posttransfection (p.t.), no obvious differences in the numbers of fluorescent cells were observed, indicating comparable transfection efficiencies among all the viruses. By 96 h p.t., most of the cells transfected with vAcWT bacmid DNA showed fluorescence, while the number of fluorescent cells transfected with v38K:SM2, v38K:SM3, or v38K:SM4 bacmid DNA did not increase from 24 to 96 h p.t. (Fig. 3A), indicating the inability of these viruses to spread the infection beyond the cells initially transfected. On the other hand, a slight increase in the number of fluorescent cells was observed for v38K:SM1 from 24 to 96 h p.t. (Fig. 3A), suggesting that limited infectious BVs were produced from the initially transfected cells. The ability of v38K:SM1 to produce infectious BV was analyzed by 50% tissue culture infective dose (TCID_50_) endpoint dilution assay using virus supernatants harvested from transfected cells at 96 h p.t. As shown in Fig. 3B, the total number of infectious BVs generated by v38K:SM1 was approximately 15,000-fold lower than that generated by vAcWT (*P* < 0.01).

Plaque assays were performed, and plaques were photographed at 96 h p.t. to further compare the spreading of virus to neighboring cells among these viruses. Plaques produced by v38K:SM2, v38K:SM3, and v38K:SM4 consisted of single cells (Fig. 3A), indicating that these viruses were unable to spread. Although larger plaques consisting of several cells were generated by v38K:SM1 (Fig. 3A), they were significantly smaller than those from vAcWT bacmid DNA-transfected cells (*P* < 0.0001) (Fig. 3C).

The expression of 38K in the 38K mutants in virus bacmid DNA-transfected cells was confirmed by immunoblotting with anti-38K antibodies, and representative immunoblots are shown in Fig. 2B (left panels). The quantitative results for protein expression levels are shown in Fig. 2C (left panel) and indicate that the expression of the modified 38K was at levels similar to those for the wild-type 38K in a *gp64* knockout virus (vGP64KO) which lacks infectivity in insect cells (37, 38) and was used as a noninfectious control virus. Thus, the above results demonstrated that the conserved sequence motifs of the HAD superfamily in 38K play an important role in viral replication.

**FIG 4:**
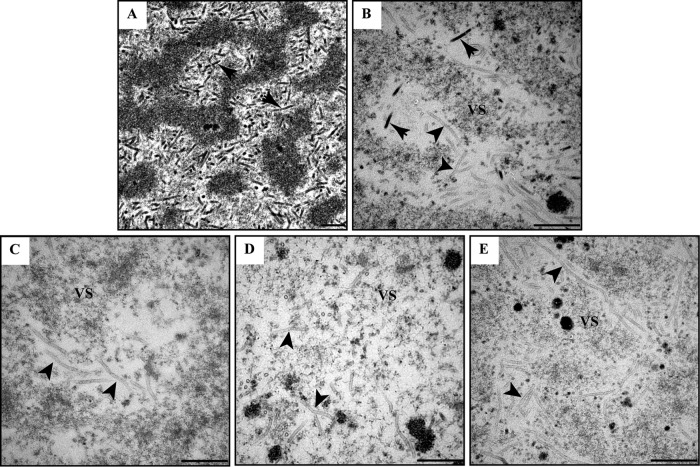
Mutation of HAD motifs in 38K affects nucleocapsid assembly. Transmission electron microscopy analysis of cells transfected with bacmids of vAcWT (A), v38K:SM1 (B), v38K:SM2 (C), v38K:SM3 (D), or v38K:SM4 (E) at 72 h p.t. is shown. (A) Electron-dense nucleocapsids are indicated by arrows. (B) Electron-dense nucleocapsids and electron-lucent tubules in the virogenic stroma (VS) are indicated by arrows and arrowheads, respectively. (C to E) Electron-lucent tubules in the VS are indicated by arrowheads. Scale bars represent 500 nm.

The deletion of *38K* from the AcMNPV genome resulted in no BV production and interruption of nucleocapsid assembly by producing capsid sheaths devoid of genomic DNA (32). To determine the effects of mutations in the HAD motifs of 38K on virion morphogenesis, thin sections of Sf9 cells transfected with bacmid DNA of vAcWT and 38K mutant viruses were analyzed via transmission electron microscopy (TEM). As expected, the formation of VS interspersed with rod-shaped electron-dense nucleocapsid (Fig. 4A) and ODV-containing polyhedra within the RZ (data not shown) was observed in vAcWT-transfected cells. In the cells transfected with v38K:SM1, although the VS was developed and several electron-dense nucleocapsids were observed (Fig. 4B), masses of electron-lucent tubular structures were present at the intrastromal space of the VS (Fig. 4B). ODV-embedding polyhedra were also observed in v38K:SM1-transfected cells (data not shown). On the other hand, the v38K:SM2-, v38K:SM3-, or v38K:SM4-transfected cells displayed a v38KKO-like pattern of virion morphogenesis, with development of the VS, formation of masses of electron-lucent tubular structures (Fig. 4C to E), and normal sizes of polyhedra without embedding of ODVs (data not shown). Even though v38K:SM1 produces some infectious BVs, the results from the TEM analysis indicated that all of the HAD motifs of 38K are essential for nucleocapsid assembly.

### 38K mediates the dephosphorylation of P6.9.

The baculovirus-encoded protamine-like protein P6.9 is composed predominantly of arginines (40%), serines/threonines (31%), tyrosines (13%), and glycines (9%) and is highly positively charged. In our previous study, 7 phosphorylated (no. 1 to 7) and 1 unphosphorylated (no. 0) P6.9 species resolved by AU-PAGE were observed by immunoblotting with protein samples from WT-resembling virus vPK1:FLAG-infected cells (24). Different species of P6.9 can be briefly classified into 3 groups: unphosphorylated species found in BVs (the no. 0 rung in AU-PAGE), hypophosphorylated species (rungs 1 to 4 in AU-PAGE) that may be phosphorylated by host kinases, and hyperphosphorylated species (rungs 5 to 7 in AU-PAGE) that are dependent on viral protein kinase PK1 phosphorylation (24). One phosphate group decreases the positive charge of P6.9 by one; therefore, each slower-migrating rung represents an increased phosphorylation occupancy. Since *38K* deletion or mutations in the 38K HAD motifs resulted in interruption of nucleocapsid assembly and since the phosphorylation cycle of P6.9 is essential for condensing DNA into procapsids to form nucleocapsid (24, 25, 39), it is worth assessing the potential involvement of 38K in the phosphorylation cycle of P6.9 during nucleocapsid assembly.

**FIG 5:**
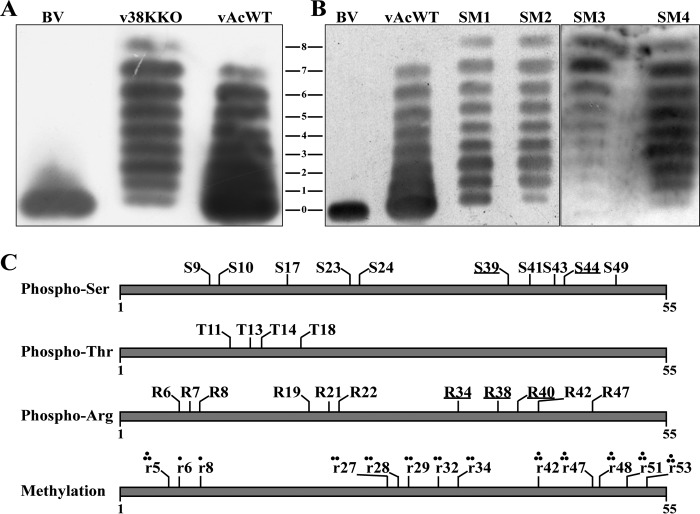
38K mediates the dephosphorylation of P6.9. (A and B) Immunoblotting for P6.9 in bacmid DNA-transfected cells. Sf9 cells were transfected with bacmid DNA of vAcWT, v38KKO, SM1 (v38K:SM1), SM2 (v38K:SM2), SM3 (v38K:SM3), or SM4 (v38K:SM4). At 48 h p.t., the transfected cells were selected using flow cytometry and were subjected to AU-PAGE for immunoblotting with the anti-P6.9 polyclonal antibodies. BV, BVs purified from the supernatants of vAcWT-infected cells. Different species of P6.9 in v38KKO or 38K mutant virus bacmid DNA-transfected cells are indicated by numbers based on their mobility ratio in AU-PAGE. (C) PTM sites of P6.9 in v38KKO-transfected cells. All of the identified phosphorylation and methylation sites of P6.9 are shown in the schematic. The phosphorylated residues are indicated by capital letters; the methylated residues are indicated by lowercase letters. The phosphorylation sites of P6.9 dephosphorylated by 38K are marked by underlining. Circles represent the mono- and dimethylation sites.

Sf9 cells transfected with bacmid DNA of v38KKO or vAcWT were sorted by flow cytometry at 48 h p.t. and subjected to AU-PAGE following immunoblotting with anti-P6.9 antibodies. As expected, only the unphosphorylated species of P6.9 was detected in BVs, and a ladder composed of 8 species of P6.9, including the unphosphorylated rung, was observed in vAcWT-transfected cells (Fig. 5A). However, 9 species of P6.9 were detected in v38KKO-transfected cells, with an additional lower-migrating rung (no. 8 in AU-PAGE) that represents a phosphorylation level higher than that of P6.9 species in vAcWT-transfected cells (Fig. 5A). This finding confirmed that some phosphorylation sites of P6.9 are unable to be dephosphorylated in the absence of 38K, suggesting that 38K mediates the dephosphorylation of P6.9 during the viral replication cycle.

To further investigate whether the HAD motifs of 38K are involved in dephosphorylation of P6.9, the phosphorylation states of P6.9 in cells transfected with each 38K mutant virus bacmid DNA were analyzed. As shown in Fig. 5B, the more-hyperphosphorylated species of P6.9 (no. 8 in AU-PAGE) was also detected in cells transfected with each 38K mutant virus bacmid DNA, which was similar to the pattern detected in v38KKO-transfected cells. Although the P6.9 species in v38K:SM1-transfected cells has the same phosphorylation pattern as in v38KKO, the amount of the unphosphorylated species appeared to be increased in v38K:SM1-transfected cells compared to that with v38KKO or other 38K mutant viruses (Fig. 5B), which may be due to the production of limited infectious BVs. These results indicated that the HAD motifs of 38K are required for the dephosphorylation of P6.9 during virus replication.

### Five phosphorylation sites at the C terminus of P6.9 identified to be dephosphorylated by 38K.

The phosphorylation sites of different P6.9 species were analyzed in Sf9 cells infected with WT-resembling virus vPK1:FLAG by MS in our previous study (24). A total of 22 residues, including 8 serines, 5 threonines, and 9 arginines, were determined to be phosphorylated in rungs 1 to 7 of P6.9 species separated by AU-PAGE, whereas none was identified in the BVs or no. 0 P6.9 species in AU-PAGE (24).

To determine the specific phosphorylated residues that were dephosphorylated by 38K, the phosphorylated species of P6.9 in v38KKO bacmid DNA-transfected Sf9 cells were analyzed by MS utilizing both collision-induced dissociation and electron transfer dissociation fragmentation techniques, as previously described (24). The results showed that a total of 25 residues of P6.9 were phosphorylated in v38KKO bacmid DNA-transfected cells, among which 10 were serine residues (S9, S10, S17, S23, S24, S39, S41, S43, S44, and S49), 4 were threonine residues (T11, T13, T14, and T18), and 11 were arginine residues (R6, R7, R8, R19, R21, R22, R34, R38, R40, R42, and R47) (Fig. 5C and Table 1). In line with a previous study in which the phosphorylation sites of P6.9 in vPK1:FLAG-infected cells were determined (24), the phosphoRS probabilities of these sites are all above 75 (Table 1), and these phosphorylated sites were confirmed by manual validation based on site-determining ions in tandem MS (MS/MS) spectra, indicating that these sites were indeed phosphorylated. Therefore, by comparison with the previous results, 5 new phosphorylation sites (R34, R38, S39, R40, and S44) of P6.9 were revealed in v38KKO-transfected cells and were thought to be specifically dephosphorylated by 38K. However, 2 phosphorylation sites (T45 and R48) observed in vPK1:FLAG-infected cells previously (24) were not detected in the current study, and the reason is unknown.

**TABLE 1:** P6.9 phosphorylation site mapping data

Sequence of identified peptide^*a*^	Site(s)	PhosphoRS probability(ies)
RRRRRSStGTtY	T11, T14	86.8, 86.8
RrSrSRSSTGrRSY	R38, R40, R47	99.9, 99.9, 99.9
RRSRSrSSTGRRSY	R42	100.0
RRRrrSSTGTTY	R7, R8	99.2, 100.0
RRrRRSSTGTTY	R6	87.2
RRRRrSSTGTTY	R8	97.7
GSTRRRrSSGY	R22	89.6
GSTRRrrSSGY	R21, R22	96.4, 99.8
RRRRRSStGTTY	T11	91.5
RRRRRSSTGtTY	T13	75.7
RRrRRSSTGTTY	R7	90.5
RRSRsrSSTGRRSY	S41, R42	93.2, 93.2
GSTrRRRSsGY	R19, S24	80.3, 82.7
GSTRRRRssGYRRRPGRPRTY	S23, S24	98.9, 98.9
RRRRrSSTGTTY	S9	81.5
RRSRsRsSTGRRSY	S41, S43	99.9, 99.9
RRRRRssTGTTY	S9, S10	100.0, 100.0
GSTRRRRssGY	S23, S24	97.8, 99.3
RRsRsRSsTGRRSY	S39, S41, S44	100.0, 100.0, 99.6
RRRRRsStGTTY	S9, T11	95.9, 94.6
RRRRRSSTGtTY	T13	76.3
GSTRRRrSSGY	R22	89.5
RRRRrSSTGTTY	R8	99.4
GSTRRrrSSGY	R21, R22	96.6, 99.9
RRRrRSSTGTTY	R7	91.8
GSTrRRrSSGY	R19, R22	99.0, 95.0
RRRRRSsTGTTY	S10	85.7
GSTRRRRsSGY	S23	87.5
GSTRRRRssGY	S23, S24	99.6, 99.8
RRsRsRSsTGRRSY	S39, S41, S44	100.0, 100.0, 94.3
GSTRRRRSsGY	S24	90.8
RRRRRSStGTTY	T11	99.4
GStRRRRSSGY	T18	100.0
GSTRRrRSSGYRRRPGRPrTY	R21, R34	96.4, 83.1
RrsrSRSSTGRRSY	R38, S39, R40	88.9, 99.5, 82.7
RRSRSRsSTGRRsY	S43, S49	99.9, 89.5

a The phosphorylated residues are indicated by lowercase letters.

In addition to the phosphorylation modifications, the presence of other PTMs of P6.9 was also investigated in raw MS data obtained from the v38KKO-transfected cells. No acetylated sites were detected in the P6.9 species, which is consistent with a previous study (24). Many methylated sites were identified, as before, including monomethylation and dimethylation: only R6 and R8 were monomethylated; R27, R28, R29, R32, and R34 were dimethylated; and R5, R42, R47, R48, R51, and R53 showed both states (Fig. 5C).

### A phosphorylation-mimic mutant of P6.9 impairs nucleocapsid assembly.

To investigate the role of dephosphorylation of P6.9 mediated by 38K in the virus life cycle, a phosphorylation-mimic mutant virus (vP6.9:5E) and a phosphorylation-deficient mutant virus (vP6.9:5A) were constructed, as described in Materials and Methods. The phosphorylation sites in P6.9 that were postulated to be dephosphorylated by 38K were mutated to glutamic acids in vP6.9:5E to mimic the phosphorylation of P6.9 or were mutated to alanines in vP6.9:5A to simulate the dephosphorylation of P6.9 (Fig. 2A).

**FIG 6:**
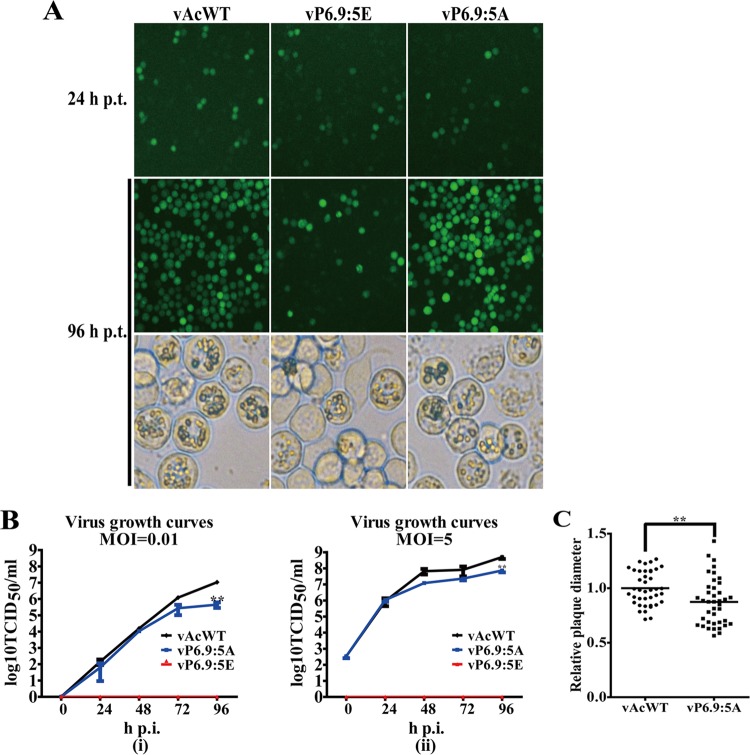
A phosphorylation-mimic mutant of P6.9 impairs virus replication. (A) Sf9 cells were transfected with vAcWT, vP6.9:5E (phosphorylation-mimic mutant), or vP6.9:5A (phosphorylation-deficient mutant) bacmid DNA. At the indicated time points, cells were visualized by fluorescence microscopy for infection. Light microscopy images show the formation of OBs in the transfected cells at 96 h p.t. (B) Virus growth curves. Sf9 cells were infected with vAcWT or vP6.9:5A at an MOI of 0.01 (i) or 5 (ii) TCID_50_/cell or all the supernatants from vP6.9:5E-transfected cells at 96 h p.t. At the indicated time points, BV titers were determined by TCID_50_ endpoint dilution assays. (C) Plaque diameters in vAcWT- or vP6.9:5A-infected cells. A total of 40 plaques were measured in vAcWT- or vP6.9:5A-infected cells. Plaque size differences were analyzed by Student's *t* test with GraphPad Prism software. Bars indicate the medians for samples. **, *P* < 0.01.

The expression of the mutated P6.9 was confirmed by immunoblotting using protein samples prepared from vP6.9:5E or vP6.9:5A bacmid DNA-transfected cells (Fig. 2B, right panels), and the expression levels of the mutated P6.9 in vP6.9:5A- or vP6.9:5E-transfected cells were compared with those of a wild-type P6.9 in vAcWT or vGP64KO, respectively (Fig. 2C, right panel). The results indicated that the expression levels of the mutated P6.9 are at levels similar to those of the wild-type P6.9 in their respective control virus (Fig. 2C, right panel). Because no infectious virions were produced by vP6.9:5E and vGP64KO, the expression levels of P6.9 in vP6.9:5E- or vGP64KO-transfected cells are significantly lower than those in vP6.9:5A- or vAcWT-transfected cells (Fig. 2C, right panel). Transfection of bacmid DNA of the recombinant viruses in Sf9 cells was monitored by microscopy and analyzed by virus growth curve analyses and plaque assays. Replication of vP6.9:5E in the cells was similar to that of v38KKO, including single-cell infection, as indicated by eGFP expression examined by fluorescence microscopy, OB formation in the infected cells as observed under light microscopy, and undetectable infectious BV production as shown by virus growth curve analysis (Fig. 6A and B). For the cells transfected with vAcWT or vP6.9:5A bacmid DNA, the number of fluorescent cells increased dramatically from 24 to 96 h p.t., and OBs were observed in many infected cells, indicating that complete virus replication cycles occurred (Fig. 6A). Virus growth curve analyses showed that the BV titer of vP6.9:5A at 96 h postinfection was 10-fold lower than that of vAcWT at a multiplicity of infection (MOI) of 5 (*P* < 0.01) and 25-fold lower at an MOI of 0.01 (*P* < 0.01) (Fig. 6B). Virus plaques produced by vP6.9:5E consisted of single cells, whereas plaques from vAcWT or vP6.9:5A bacmid DNA-transfected cells contained a number of fluorescent cells (data not shown). A relative quantitative analysis of the sizes of virus plaques showed that plaques produced by vP6.9:5A were significantly smaller than those produced by vAcWT (*P* < 0.01) (Fig. 6C).

**FIG 7:**
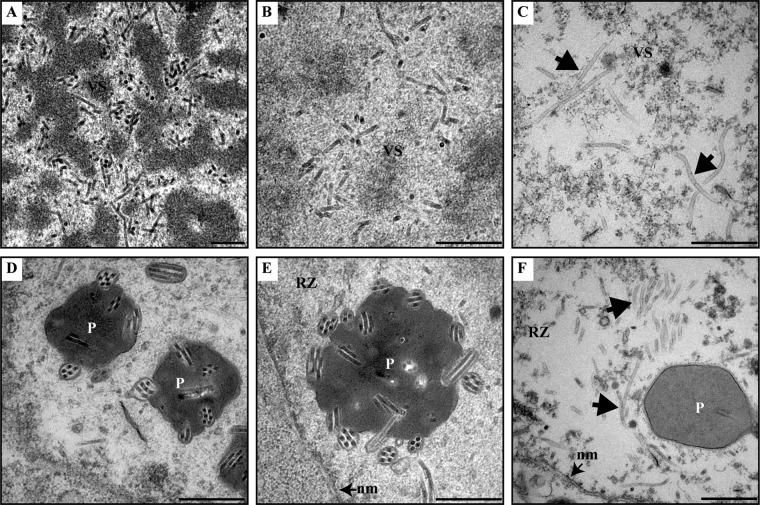
A phosphorylation-mimic mutant of P6.9 interrupts nucleocapsid assembly. vAcWT-transfected (A and D), vP6.9:5A-transfected (B and E), or vP6.9:5E-transfected (C and F) cells at 72 h p.t. are shown. (A and B) Rod-shaped electron-dense nucleocapsids are observed in the VS. (D and E) ODVs and polyhedra containing ODVs are found within the RZ. (C and F) Abnormally long, electron-lucent tubules (indicated by arrows) can be found in the VS (C) and in the RZ (F). VS, virogenic stroma. P, polyhedra. RZ, ring zone. nm, nuclear membrane. The bars represent 500 nm.

To assess the effects of mutations in P6.9 on viral nucleocapsid assembly, thin sections of cells transfected with bacmid DNA were analyzed by TEM. The vP6.9:5A-transfected cells showed results similar to those with vAcWT-transfected cells (Fig. 7A and D), including the presence of VS containing rod-shaped electron-dense nucleocapsids (Fig. 7B) and enveloped ODVs containing multiple nucleocapsids within the RZ or embedded in polyhedra (Fig. 7E). In contrast, aberrant capsids or electron-lucent tubular structures were observed at the intrastromal space of the VS and within the RZ of cells transfected with vP6.9:5E (Fig. 7C and F). These results revealed that the phosphomimetic mutation of P6.9 impaired the assembly of nucleocapsids.

## DISCUSSION

Viral genomes are condensed and packaged into capsids by multiple mechanisms during encapsidation (40–42). Essential to this process is neutralization of the negative electrostatic charge on the phosphate groups of the nucleotide backbone by positively charged ions, such as divalent cations (43) or other small basic cationic proteins (44). Baculovirus encodes a protamine-like small basic protein, P6.9, which is rich in arginine (40%) and serine/threonine (31%) residues (45). Evidence indicates that P6.9 is prevented from binding to DNA as a result of phosphorylation modification of arginine and serine residues (22, 46, 47). During viral assembly, P6.9 is dephosphorylated for neutralization of viral DNA and condensing DNA into the viral capsid (25, 48). A previous study showed that multiple phosphorylation species of P6.9 were detected in virus-infected cells, but only the unphosphorylated species was presented in BVs (24), indicating that P6.9 undergoes phosphorylation and dephosphorylation cycles during virus replication. However, phosphatases of the virus or host that are responsible for the dephosphorylation of P6.9 during nucleocapsid assembly have not yet been determined. Here, our data suggest that baculovirus 38K may mediate dephosphorylation at 5 specific sites (R34, R38, S39, R40, and S44) at the C terminus of P6.9 and that dephosphorylation of these sites is essential for packaging of genomes in to capsids.

*38K* is one of the 38 core genes of baculovirus (49), and its deletion results in interruption of viral genome encapsidation (32, 50). Evolutionary genomics analysis showed that 38K belongs to the HAD superfamily, which is characterized by HAD motifs. Our current study confirmed that the HAD motifs of 38K were important for nucleocapsid assembly, since mutation of any motif resulted in production of abnormal electron-lucent tubular structures devoid of DNA content in virus-transfected cells (Fig. 4). Although these results imply that 38K may function as a viral phosphatase and that the enzyme activity may participate in nucleocapsid assembly, *in* *vitro* experiments to detect the phosphatase activity using purified 38K protein were unsuccessful (data not shown), suggesting that appropriate substrates or experimental conditions are critical. Among the members of the HAD superfamily, HAD motifs II and III contribute to the stability of the reaction intermediates of the hydrolysis reaction, and motif IV along with motif I is required for coordinating a magnesium ion in the active site (31). The mutations of these motifs in MDP-1, phosphoserine phosphatase (PSPase), and ATPase resulted in a loss of activity (33, 51, 52). Hence, the mutation of HAD motifs in 38K might alter its enzymatic activity and result in functional loss of 38K. Although a few nucleocapsids with DNA content were detected (Fig. 4B), and infectious BVs were produced (Fig. 3) in motif I-mutated virus bacmid DNA-transfected cells, the nucleocapsid assembly was severely impaired, and the BV production was 15,000-fold lower than that of the control virus. We speculate that the active center of 38K, which should have resided in motif I, may have partially been replaced by another conserved amino acid during evolution. 38K molecules have been shown to interact with each other as homo-oligomers (53), which suggests that 38K may function as a homodimer, unlike other HAD members.

As P6.9 undergoes phosphorylation-dephosphorylation cycling during nucleocapsid assembly, the potential involvement of 38K in regulating the dephosphorylation of P6.9 was investigated in this study. The deletion of *38K* or the mutation of the HAD motifs in 38K resulted in the production of a more phosphorylated species of P6.9 (no. 8 in AU-PAGE), as detected by AU-PAGE (Fig. 5A and B). The phosphorylation sites of P6.9 that are dephosphorylated by 38K were then determined by MS. Compared to the phosphorylation sites detected in the cells infected with the WT-resembling virus (24), five new sites (R34, R38, S39, R40, and S44) were identified (Fig. 5C) in v38KKO-transfected cells and thus were considered to be the sites mediated by 38K. The possible structure of P6.9 was predicted by using the online tool I-IASSER for protein and function predictions (https://zhanglab.ccmb.med.umich.edu/I-TASSER/) (54), and the result showed that the R38, S39, R40, and S44 sites of P6.9 are clustered and the R34, R38, R40, and S44 sites are on the surface of P6.9 (data not shown). The spatial distribution of these sites possibly makes them accessible to 38K. However, these sites have a low degree of conservation in the P6.9 homologs among other baculoviruses (55). In addition, there might be other phosphorylation sites of P6.9 in the cells infected with the WT-resembling virus that are dephosphorylated by 38K and may still be present in the v38KKO-transfected cells, but these sites were unable to be detected by this method; thus, the dephosphorylation sites mediated by 38K may include more than the 5 sites identified in the current study. Meanwhile, the HAD motifs in 38K are involved in P6.9 dephosphorylation, suggesting that other phosphatases from the haloacid dehalogenase superfamily which process the HAD motifs may participate in the dephosphorylation of P6.9. However, the 38K family, which might have arisen from the MDP-1/FkbH family, is much smaller and shows a very unusual phyletic pattern that is found only in viruses (31), suggesting that phosphorylation of P6.9 at these 5 sites might be exclusively dephosphorylated by viral 38K. On the other hand, the unphosphorylated species of P6.9 present in cells transfected with *38K*-deleted or -mutated virus bacmid DNA (Fig. 5A and B) indicated that other phosphatases take part in the regulation of dephosphorylation of other sites in P6.9. In addition, both *38K* and *p6.9* are viral late genes. Their transcripts were both expressed from 12 h postinfection (45, 56). Although 38K does not contain a nuclear localization signal by prediction, it localized to the nuclei of transfected cells (53) and localized mainly to the center of the nucleus in the late phase during virus infection (32). On the other hand, the majority of the P6.9 localized near the inner nuclear membrane, whereas a lower concentration of P6.9 was observed in the VS (23). Therefore, dephosphorylation of P6.9 mediated by 38K might take place in the nucleus during virus infection. Even though 38K mediates the dephosphorylation of P6.9, attempts to detect the interaction between 38K and P6.9 by yeast two-hybrid experiments (50) and coimmunoprecipitation assays (data not shown) were unsuccessful. We speculate that the interaction between 38K and P6.9 is transient and thus difficult to be detected.

Three of the 5 phosphorylation sites (R38, S39, and R40) dephosphorylated by 38K are in a 3-arginine-serine (RS) repeat region (38RSRSRS43) of P6.9 (Fig. 5C). These sites may be phosphorylated by host kinases, as the RS repeats in protamine are potential phosphorylation targets of arginine/serine protein-specific kinase 1 (57) and topoisomerase I (20). The phosphorylation-deficient mutant virus of P6.9 (vP6.9:5A) produced fewer infectious BVs and smaller plaques (Fig. 6B and C), which may have resulted from fewer phosphate groups added to P6.9. Evidence suggests that newly synthesized P6.9 is transiently phosphorylated in virus-infected cells prior to participation in nucleocapsid assembly (22); thus, the phosphorylation of the sites mutated to alanines in vP6.9:5A is beneficial for virus replication. Therefore, nonphosphorylation of these sites in vP6.9:5A resulted in reduced BV production. Similarly, in a previous study in which other phosphorylation sites of P6.9 were mutated to alanines, the viral infectivity was severely impaired (24). On the other hand, the phosphorylation-mimic mutant virus (vP6.9:5E) led to a failure of viral DNA encapsidation (Fig. 7). PTMs regulate protein conformation as well as protein-protein interactions, and the phosphorylation of protamine has been suggested to disturb intraprotamine and interprotamine interactions (58, 59). Therefore, the phosphorylation-mimic mutant of P6.9 may disrupt the tightness of P6.9 complexes and interfere with the interaction of P6.9 with DNA due to electrostatic repulsion, resulting in unsuccessful condensation of viral DNA. Moreover, a previous study showed that the dephosphorylation of P6.9, at least at the C terminus (aa 29 to 55), occurred at the surface of the electron-dense stoma matte of the VS and at what appeared to be nucleocapsids undergoing assembly, indicating that the C terminus of P6.9 undergoes dephosphorylation during nucleocapsid assembly (23). Thus, the inability to dephosphorylate the phosphorylation sites located at the C terminus of P6.9, which was the case for the phosphorylation-mimic mutant in the present study, resulted in unsuccessful nucleocapsid assembly.

Baculoviruses are not the only double-stranded DNA (dsDNA) viruses that use protamine-like proteins to condense their genome. WSSV encodes a similar protein named VP15, which is major nucleocapsid protein and has been shown to bind both AcMNPV and WSSV DNAs (60). The phosphorylation of P6.9 in baculovirus may be important for the decondensation and ejection of the viral genome into the cell nucleus (25), whereas the phosphorylated species of VP15 have not been found yet (60). This could imply that VP15 may use a different mechanism for releasing DNA. The P64 protein of Spodoptera frugiperda ascovirus 1a (SfAV1a) contains 14 copies of a highly basic, tandemly repeated motif rich in arginine and serine residues at its C terminus. It has been shown that P64 binds SfAV1a DNA and is incorporated into the SfAV1a DNA core during virion assembly, supporting the hypothesis that P64 is involved in packaging viral DNA (61). Whether nascent P64 is phosphorylated is unknown, but it is unphosphorylated in the mature virion (61). Adenoviruses also encode a highly basic protein called protein VII that binds and condenses DNA (62). In addition, protein VII has PTMs, including phosphorylation, that contribute to its chromatin localization and hinder chromatin association of host proteins, leading to abrogated immune responses (63). P6.9 was located primarily within the region of marginalized host chromatin induced by baculovirus infection (23), suggesting that P6.9 may adopt a strategy similar to that of protein VII to alter host chromatin dynamics. Taken together, the data indicate that although basic proteins encoded by different viruses can be either phosphorylated or unphosphorylated, only unphosphorylated species of basic proteins are incorporated into mature virions, where they presumably assist in neutralizing negative electrostatic charges conferred by phosphate groups in DNA. Therefore, for dsDNA viruses using basic proteins to condense and package DNA into nucleocapsids, it is necessary that phosphorylated basic proteins, if any, be dephosphorylated during nucleocapsid assembly.

It should be noted that in addition to mediating the dephosphorylation of P6.9, 38K may participate in nucleocapsid assembly in other ways. 38K is a nucleocapsid-associated protein and interacts with other capsid proteins, such as VP39, VP1054, and VP80 (50). It is possible that these interactions may be required for nucleocapsid assembly, and the deletion or mutation of *38K* may disrupt these interactions and thus result in a failure of nucleocapsid assembly. For example, the nucleocapsid assembly was interrupted and the distribution of 38K was intensively changed with the deletion of the gene encoding 38K-interacting protein VP1054 (64); thus, the function of 38K may be abolished due to its aberrant localization.

In summary, our current study demonstrated that the viral nucleocapsid-associated protein 38K mediates the dephosphorylation of 5 specific residues at the C terminus of P6.9. The phosphorylation of these sites promotes virus replication, while their dephosphorylation is essential for nucleocapsid assembly in the AcMNPV life cycle. This suggests, at least partially, a novel function and mechanism of 38K in the virus life cycle. To further elucidate how phosphorylation and dephosphorylation cycles of P6.9 are regulated to facilitate genome ejection and encapsidation, further experimentation is needed to identify other phosphatases or kinases that act on P6.9.

## MATERIALS AND METHODS

### Cells and viruses.

The Sf9 (Spodoptera frugiperda IPLB-Sf21-AE clonal isolate 9) insect cells were cultured at 27°C in Grace's medium (Invitrogen) supplemented with 10% fetal bovine serum, 100 μg/ml penicillin, and 30 μg/ml streptomycin. The AcMNPV *38K*-knockout bacmid vAc^38K-KO^ (defined as b38KKO in the present study), the *38K*-knockout virus with *polyhedrin* and *egfp* genes vAc^38K-KO-PH-GFP^ (defined as v38KKO in the present study), and a control virus resembling the WT AcMNPV vAc^PH-GFP^ (defined as vAcWT in the present study) were constructed in our previous study (32). The *p6.9*-knockout recombinant bacmid bP6.9KO was also constructed previously by Liu et al. (23).

### Bioinformatics analyses.

The conserved domains in AcMNPV 38K were predicted from the NCBI Conserved Domain Search database (https://www.ncbi.nlm.nih.gov/Structure/cdd/wrpsb.cgi) (34), and the homologs of 38K were searched against the NCBI protein database (https://blast.ncbi.nlm.nih.gov/Blast.cgi?PROGRAM=blastp&PAGE_TYPE=BlastSearch&LINK_LOC=blasthome) with the BLAST algorithm. Multiple-sequence alignments of 38K were carried out with Clustal X (65) with default settings and were edited with GeneDoc (66). The 38K homologs selected for sequence alignments (Fig. 1A) from the family Baculoviridae were as follows: the group I alphabaculoviruses AcMNPV, Bombyx mori NPV, and Orgyia pseudotsugata MNPV; the group II alphabaculoviruses Agrotis segetum NPV, Spodoptera exigua MNPV, and Lymantria dispar MNP; the betabaculoviruses Agrotis segetum granulovirus (GV), Cydia pomonella GV, and Spodoptera litura GV; the deltabaculovirus Culex nigripalpus NPV; and the gammabaculoviruses Neodiprion abietis NPV, Neodiprion lecontei NPV, and Neodiprion sertifer NPV. In addition, 38K homologs from the families Nudiviridae and Polydnaviridae were also selected for multiple-sequence alignment, including the family Nudiviridae viruses Oryctes rhinoceros nudivirus, Kallithea virus, Nilaparvata lugens endogenous nudivirus, Gryllus bimaculatus nudivirus, Helicoverpa zea nudivirus, Tipula oleracea nudivirus, and Penaeus monodon nudivirus and the family Polydnaviridae viruses Microplitis demolitor bracovirus and Cotesia congregate bracovirus.

### Construction of recombinant viruses.

To dissect the function of 38K in nucleocapsid assembly, the four highly conserved motifs of the HAD superfamily in 38K, i.e., D^140^xD^142^ motif I, S^177^ motif II, K^251^ motif III, and D^275^D^276^ motif IV, were mutated to A^140^xA^142^, A^177^, A^251^, and A^275^A^276^ (Fig. 2A) individually by a previously described method (67). To this end, a 1,522-bp fragment containing the WT *38K* gene with its own promoter and poly(A) signal was first PCR amplified using primers Ac9855 and Ac98310 (32) and was ligated to the pMD18-T vector (TaKaRa) using PstI and XbaI sites to generate T-38KPOA2. Four sets of primers (all primers used in this study are presented in Table 2) were then used to construct the recombinant plasmids T-38KSM1, T-38KSM2, T-38KSM3, and T-38KSM4 using T-38KPOA2 as a template. Primers 98517Ft, 98518Fs, 98313Rt, and 98314Rs were used to introduce A^140^xA^142^ in 38K; primers 98519Ft, 98520Fs, 98315Rt, and 98316Rs were used to introduce A^177^ in 38K; primers 98521Ft, 98522Fs, 98317Rt, and 98318Rs were used to introduce A^251^ in 38K; and primers 98523Ft, 98524Fs, 98319Rt, and 98320Rs were used to introduce A^275^A^276^ in 38K. The 38KSM1, 38KSM2, 38KSM3, and 38KSM4 fragments were released from the T vectors by PstI/XbaI digestion and were ligated to pFB1-PH-GFP (32) to generate donor plasmids pFB-38KSM1-PG, pFB-38KSM2-PG, pFB-38KSM3-PG, and pFB-38KSM4-PG, respectively. The donor plasmids were then transformed into Escherichia coli DH10B cells containing helper plasmid pMON7124 and the *38K*-knockout bacmid b38KKO to generate 38K site-mutated viruses v38K:SM1, v38K:SM2, v38K:SM3, and v38K:SM4, respectively.

**TABLE 2:** Primers used in this study

Primer	Sequence
98517Ft	5′-GTGTTTGCATTGGCAAGCACTCTCATAACCGAAGAGGAGC-3′
98518Fs	5′-ACTCTCATAACCGAAGAGGAGC-3′
98313Rt	5′-GCTTGCCAATGCAAACACGACCACGTGCGGAAATCCCCAC-3′
98314Rs	5′-GACCACGTGCGGAAATCCCCAC-3′
98519Ft	5′-TTGTGGGCATATGGCAGTAGAGATCATGTAGCACACTCGA-3′
98520Fs	5′-AGAGATCATGTAGCACACTCGA-3′
98315Rt	5′-ACTGCCATATGCCCACAAGACTAAAACGCAACCCATCTCG-3′
98316Rs	5′-GACTAAAACGCAACCCATCTCG-3′
98521Ft	5′-ATACCCGCATCGCCAAAAATTGTAATTAAATATTTGAGCG-3′
98522Fs	5′-ATTGTAATTAAATATTTGAGCG-3′
98317Rt	5′-TTTTGGCGATGCGGGTATATTGTTGTCGGATCGATGATTG-3′
98318Rs	5′-ATTGTTGTCGGATCGATGATTG-3′
98523Ft	5′-CTTGCCGCAGCACTGCCAACTAATAACTACGCGTACGATT-3′
98524Fs	5′-ACTAATAACTACGCGTACGATT-3′
98319Rt	5′-TGGCAGTGCTGCGACAAGTGTGATCGACTTGAAAAAGTTT-3′
98320Rs	5′-TGTGATCGACTTGAAAAAGTTT-3′

To investigate the role of the phosphorylation cycle of P6.9 in nucleocapsid assembly, a phosphorylation-deficient mutant virus (vP6.9:5A) and a phosphorylation-mimic mutant virus (vP6.9:5E) were constructed. In brief, the *p6.9* mutants with 5 residues (arginines at position 32, 38, and 40 and serines at position 39 and 44) mutated to alanines (A32A38A39A40A44) or glutamic acids (E32E38E39E40E44) were generated by total gene synthesis technology and then cloned into plasmid pUC19 (Ruibiotech). The mutants of *p6.9* were cloned into pUC18-Pro-SV40 (24), which contains the native promoter of *p6.9*, to generate pUC18-Pro-P6.9:5A-SV40 or pUC18-Pro-P6.9:5E-SV40. Pro-P6.9:5A-SV40 or Pro-P6.9:5E-SV40 was released from the plasmid described above and inserted into pFB1-PH-GFP to generate the donor plasmid pFB-P6.9:5A-PG or pFB-P6.9:5E-PG. Donor plasmid pFB-P6.9:5A-PG or pFB-P6.9:5E-PG then was transformed into DH10B cells containing pMON7124 and the *p6.9*-knockout bacmid bP6.9KO to generate the recombinant virus vP6.9:5A or vP6.9:5E.

All of the constructs were confirmed by PCR analysis and DNA sequencing. Bacmid DNAs of recombinant viruses were electroporated into DH10B cells and screened for tetracycline sensitivity to remove the helper plasmid. Extrapure bacmid DNAs were then extracted and purified with the Large-Construct kit (Qiagen) and quantified by optical density measurement.

### Viral growth curve analyses and plaque assay.

Bacmid DNA transfection and virus infection were performed as previously described (32). Specifically, Sf9 cells (1.0 × 10^6^ cells/35-mm-diameter dish) were transfected with 1 μg bacmid DNAs of recombinant viruses using Lipofectin (Invitrogen) or infected with viruses at an MOI of 5 or 0.01. The viral inocula were allowed to be adsorbed by the cells for 5 h upon transfection or 1 h upon infection at 27°C and were then replaced with fresh medium. Time zero was defined as the time when fresh medium was added. The supernatants of transfected or infected cells were harvested at the indicated time points, and the titers were determined by TCID_50_ endpoint dilution assay on Sf9 cells (68).

Plaque assays were performed as previously described (32). In brief, a total of 2.0 × 10^6^ cells in a monolayer were transfected separately with 0.01 μg bacmid DNA of recombinant viruses. At 120 h p.t., plaques were photographed, and the sizes of plaques were measured.

### TEM analysis.

Sf9 cells (1.0 × 10^6^) transfected with bacmid DNAs of recombinant viruses were harvested at 72 h p.t. and pelleted at 2,000 × *g* for 10 min. The cells were subjected to TEM as previously described (32). Ultrathin sections were observed with a JEM 1400 transmission electron microscope at an accelerating voltage of 120 kV.

### Flow cytometry.

Sf9 cells transfected with bacmid DNA or infected with recombinant viruses were harvested at 48 h after transfection or infection. The cells were washed twice with phosphate-buffered saline (PBS) and pelleted by centrifugation at 1,000 × *g* for 10 min. The supernatants were removed, and the cells were resuspended with PBS at approximately 1.0 × 10^7^ cells/ml. The transfected or infected cells were selected using flow cytometry (MoFlo XDP; Beckman Coulter, USA) based on green fluorescence, as previously described (24).

### AU-PAGE.

To separate the different phosphorylated species of P6.9, AU-PAGE was performed as previously described (23). For BV samples, supernatants collected from vAcWT-infected Sf9 cells were purified by sucrose gradient centrifugation and resuspended in 0.1 M Tris-EDTA (69). The cells selected by flow cytometry or the purified BVs were denatured with AU gel sample buffer and were resolved by AU-15% PAGE (26).

### Immunoblotting.

To detect the phosphorylation pattern of P6.9, total proteins resolved by AU-PAGE were transferred onto a 0.22-μm polyvinylidene fluoride membrane (Millipore) and immunoblotted with anti-P6.9 antibodies as described by Li et al. (24). To detect the expression of 38K or P6.9 in recombinant virus-transfected cells, the pelleted cells were resuspended in PBS with an equal volume of 2× protein sample buffer (0.25 M Tris-HCl [pH 6.8], 4% sodium dodecyl sulfate, 20% glycerol, 10% 2-mercaptoethanol, 0.02% bromophenol blue) and boiled at 100°C for 10 min. The samples were resolved by SDS-PAGE and transferred onto a 0.22-μm polyvinylidene fluoride membrane for immunoblotting with anti-38K polyclonal antibodies (1:500), anti-P6.9 polyclonal antibodies (1:1,000), or antiactin monoclonal antibodies (1:2,000; Abmart) and then quantified with a Gel-Pro analyzer 4. Three independent experiments were performed with actin as an internal reference protein.

### MS.

To determine the phosphorylation sites of P6.9 in v38KKO bacmid DNA-transfected cells, P6.9 phosphorylated species were separated by AU-15% PAGE as described above and stained with Coomassie brilliant blue (Sigma). The preparation of MS samples was adopted from a previous study with modifications (24). In brief, the protein bands of phosphorylated P6.9 were excised from the gel and were subjected to in-gel digestion with chymotrypsin at a final concentration of 0.05 μg/μl (MS grade; Thermo Scientific) overnight at 37°C as previously described but without the reduction and alkylation steps (70). Peptides extracted from each digested species were pooled, desalted, and purified with Pierce graphite spin columns (Thermo Scientific). The PTMs of these peptides were determined with a hybrid linear ion trap-Orbitrap mass spectrometer (LTQ Orbitrap Elite; Thermo Scientific), as described by Li et al. (24).

A database search was performed with Proteome Discoverer 1.3 (Thermo Scientific) using the SEQUEST program (71) with the amino acid sequence of AcMNPV P6.9 with search parameters set as previously described (24). The phosphoRS algorithm was also employed to evaluate the accuracy of phosphorylation site localizations (72). For each phosphorylation site, a phosphoRS probability above 75 indicated that the site actually was phosphorylated; these phosphorylated sites were confirmed by manual validation based on the presence and intensity of site-determining ions in MS/MS spectra (73).
